# Compliance and non-compliance patterns among listed and non-listed ship recycling facilities: a comparative assessment of 22 facilities evaluated for the EU List

**DOI:** 10.1007/s11356-026-38259-w

**Published:** 2026-10-03

**Authors:** Newton Narciso Pereira, Camila Aparecida Maciel da Silveira, Kauã Costa dos Santos Paula, Camila Aguiar Diniz

**Affiliations:** 1 Department of Production Engineering, Federal Fluminense University (UFF), School of Industrial Engineering and Metallurgy at Volta Redonda,, Av. Dos Trabalhadores, Volta Redonda, Rio de Janeiro, Brazil; 2 Department of Production Engineering, São Paulo State University (UNESP), School of Engineering and Science, Av. Dr. Ariberto Pereira da Cunha, Guaratinguetá, São Paulo, Brazil

**Keywords:** Ship recycling, Environmental compliance, EU ship recycling list, Governance systems, Hazardous materials management, Regulatory monitoring

## Abstract

Ship recycling plays a central role in the circular economy of the maritime industry, yet significant disparities persist in safety, environmental performance, and regulatory compliance across global Ship Recycling Facilities (SRFs). This study analyzes 22 official inspection reports conducted by DNV for the European Commission under the EU Ship Recycling Regulation (EU SRR 1257/2013), comparing listed and non-listed facilities. Using a structured qualitative framework, the findings suggest that differences between listed and non-listed facilities are reflected not only in overall compliance levels but also in the nature and distribution of non-conformities across regulatory dimensions. While most facilities showed relatively high levels of conformity in infrastructure-related requirements, more pronounced differences between the groups were observed in environmental monitoring, institutional governance, traceability systems, and intertidal zone control. Non-listed SRFs showed recurring deficiencies in environmental monitoring, documentation, emergency preparedness, and hazardous waste management, whereas listed facilities generally showed stronger governance and risk-management capabilities despite minor shortcomings. A sensitivity analysis excluding facilities employing dismantling methods prohibited under the EU SRR identified two non-listed facilities with performance profiles comparable to several listed SRFs, indicating that structural eligibility alone does not account for all observed differences between the groups. The results further suggest that environmental verification, traceability, and institutional governance are among the dimensions in which recurrent differences between the two groups were observed. These findings provide new insights into patterns associated with different listing outcomes and highlight areas that may support the modernization and regulatory alignment of ship recycling facilities.

**Supplementary Information:**

The online version contains supplementary material available at https://doi.org/10.1007/s11356-026-38259-w.

## Introduction

Ship recycling constitutes an important stage in the life cycle of vessels, particularly considering the growing global demand for sustainable industrial practices, mitigation of environmental impacts, and the proper management of hazardous materials present at the end of a ship’s operational life (Ahmed 2020; Rahman et al. 2018; Rousmaniere and Raj 2007). As an increasing number of vessels reach the end of their economic life, recycling has emerged as an essential pathway for material circulation and the promotion of a circular economy (Zhou et al. 2023a). However, in contexts marked by weak regulatory frameworks, ship recycling remains associated with severe risks to human health, environmental quality, and occupational safety (Sezer et al. 2024), despite its potential to generate employment and to recover strategically valuable metal resources.

Historically, decisions regarding whether to extend a vessel’s operating life or direct it to recycling have traditionally reflected economic considerations such as fluctuations in maritime transport markets, the availability of new vessels, and maintenance costs (Cairns 2014). Since the 1990 s, the sector has exhibited a markedly asymmetric geography: while most ships are operated by owners in the Global North, most dismantling activities occur in the Global South, particularly in India, Bangladesh, and Pakistan (Dewan and Sibilia 2024). India has accounted for a substantial share of the global volume of recycled vessels and employs tens of thousands of workers (Deshpande et al. 2013), whereas Bangladesh has become a strategic hub due to its low labor costs and strong domestic steel demand (Abdullah et al. 2023). Shipbreaking yards in these regions also generate substantial quantities of e-waste owing to the presence of electronic equipment and components containing heavy metals and other hazardous substances on board vessels (Roy et al. 2022).

Despite the economic relevance of these centers, the literature consistently highlights persistent negative externalities, environmental contamination, exposure to toxic substances, and unsafe dismantling practices, particularly in beaching operations (Rahman et al. 2018). Although several developing countries benefit from low-cost steel supplied by the ship recycling industry (Kaiser 2008), increasing international pressure since the late 1990 s has been accompanied by reforms aimed at ensuring minimum safety and environmental standards (Rousmaniere and Raj 2007). In this context, multilateral regulatory frameworks such as the Basel Convention established principles governing the transboundary movement of hazardous wastes, requiring disposal only in facilities capable of ensuring “environmentally sound management” (Alter 2000; Moen 2008). The adoption of the Hong Kong International Convention for the Safe and Environmentally Sound Recycling of Ships (HKC 2009) represented further development in the international regulatory framework by outlining global guidelines for safe and environmentally responsible ship dismantling, despite ongoing criticism regarding the permissibility of dismantling operations conducted with limited infrastructure (Ahmed 2020).

Even with these regulatory advances, ship recycling within Europe remains limited due to high operating costs and stringent environmental requirements (European Union, 2023). To regulate the recycling of EU-flagged vessels and strengthen environmental and safety requirements, the European Union adopted the EU Ship Recycling Regulation (EU SRR No. 1257/2013), which established the European List of Ship Recycling Facilities (EU List), establishing that EU-flagged ships may be recycled only at facilities included in the European List (European Commission, 2013). Inclusion in the EU List requires detailed technical inspection reports and independent audits evaluating infrastructure, environmental conditions, hazardous waste control, and legal compliance (Solakivi et al. 2021). At present, the classification society DNV has conducted assessments of SRFs located outside the EU on behalf of the European Commission. Despite the regulatory importance of this mechanism, the scientific literature lacks systematic analyses of these reports, particularly concerning patterns observed between listed and non-listed facilities, the application of assessment criteria, and transparency in the decision-making process.

Given these gaps, this study addresses the following research question: Which technical, environmental, operational, and regulatory patterns documented in inspection reports are associated with different listing outcomes for SRFs evaluated for inclusion in the EU List?

To answer this question, the article conducts a comparative analysis of inspection reports submitted by SRFs during the EU List application process, aiming to identify: (i) the technical and regulatory dimensions that differentiate listed and non-listed facilities; (ii) potential differences in the application and documentation of assessment criteria; and (iii) implications for the global governance of ship recycling. By establishing comparable analytical parameters, this study contributes to a better understanding of the patterns observed across EU List assessments and contributes to international debates on minimum environmental and safety standards for sustainable ship recycling. The findings also provide a basis for discussing regulatory compliance in ship recycling as involving institutional and managerial capabilities in addition to technical requirements.

## Theoretical background

### Dismantling methods and operational implications

Understanding dismantling methods is essential for examining both operational practices and the risks associated with ship recycling. The literature identifies four main approaches: dry dock, alongside, landing, and beaching, each differing in terms of infrastructure requirements, operational costs, degree of mechanization, and environmental control (Akter and Arifuzzaman 2023). These methods are unevenly distributed across the globe, reflecting economic and regulatory asymmetries between the Global North and the Global South.

- Dry dock: this approach has historically been adopted in Europe and has become increasingly prevalent in China (Solakivi et al. 2021). It involves conducting dismantling operations within controlled and contained areas, thereby reducing the potential for pollutant releases into the surrounding environment (Xu et al. 2025). Compared with less contained dismantling practices, the use of dry docks provides more controlled operating conditions and may contribute to lower occupational accident risks (Steuer et al. 2021).
- Floating or alongside: this method is commonly adopted in China, the European Union, and the United States of America (Tola et al. 2023). It enables dismantling operations to be conducted under more controlled conditions using cranes, thereby reducing the risk of pollutant releases and accidental spills compared with the conventional beaching method (Sant’ Ana et al. 2026). Dismantling generally begins with the upper sections of the vessel and proceeds from top to bottom (Schøyen et al. 2017).
- Landing: this method is commonly used in Turkey and may offer advantages in terms of occupational safety and environmental control (Tola et al. 2023). In this method, ships are pushed or pulled onto a concrete slipway, which facilitates spill containment and subsequent clean-up operations. Typically, a mobile crane operating from the shore is used to remove sections of the hull and machinery components from the vessel. The relatively small tidal range and improved access to the hull and working area may provide operational, safety, and environmental advantages over the conventional beaching method (ElMenshawy et al. 2024).
- Beaching: according to Kakar et al. (2021), ships are beached during high tide and subsequently dismantled manually at low tide. The dismantled materials are then cut into smaller sections and segregated in storage yards located along the beach. As dismantling operations are conducted directly along the shoreline, hazardous substances and wastes from end-of-life ships, including asbestos, oily residues, and toxic metals, may leach into or be released directly into the marine environment. Beaching remains widely used in parts of South Asia, particularly in Bangladesh, India, and Pakistan, where its prevalence has been associated with lower operating costs, limited infrastructure, and historically less stringent regulatory conditions (Mathew 2022).

Examining the dismantling methods adopted by SRFs is important for interpreting differences in structural eligibility and compliance patterns under EU regulations. Although permitted under the Hong Kong Convention, beaching is not considered compatible with the requirements applicable to the recycling of EU-flagged vessels under the European Ship Recycling Regulation (European Commission, 2013) particularly because of concerns related to environmental containment, worker safety, and operational control (Dewan and Sibilia 2024). Accordingly, dismantling methods constitute an important dimension of structural eligibility when comparing listed and non-listed SRFs and are among the aspects in which differences between listed and non-listed facilities were observed across the inspection reports.

### Health, safety, and environmental (HSE) conditions

Ship dismantling activities in several developing-country contexts are associated with substantial risks to occupational health and environmental integrity (Rahman et al. 2018; Rousmaniere and Raj 2007). Workers frequently face long working hours, low wages, and insufficient training, often without adequate personal protective equipment (Sonak et al. 2008). The handling of hazardous substances, such as asbestos, PCBs, heavy metals, and hydrocarbons, has also been reported in contexts where adequate containment structures or formalized waste-disposal systems are limited or absent (Misra 2019).

Empirical studies conducted in Bangladesh and other South Asian countries have reported significant contamination of marine sediments, coastal waters, and locally cultivated food products, raising concerns regarding potential public health impacts (Deshpande et al. 2012; Hasan et al. 2020). Complementary evidence from Chinese shipbreaking yards suggests that inadequate training procedures and limitations in management systems are associated with higher occupational accident rates (Zhou et al. 2023b).

Despite the breadth of documented hazards, relatively few studies examine how HSE-related concerns are incorporated into the technical criteria applied by the European Union when assessing SRFs for inclusion in the European List. Requirements related to hazardous waste management, emergency preparedness, International Organization for Standardization (ISO) certifications, and exposure control constitute important components of the EU List assessment framework (European Union, 2016). Discrepancies between practices commonly reported in Global South facilities and the regulatory requirements applied under the EU framework are frequently observed in EU inspection reports and are among the areas in which differences between listed and non-listed facilities are observed. Commonly reported non-conformities include the lack of impermeable working surfaces, inadequate procedures for handling hazardous waste, insufficient formal training, weak effluent containment systems, and the absence of continuous environmental monitoring.

Although multilateral instruments such as the Basel Convention and the Hong Kong Convention establish normative principles for environmentally sound ship recycling, their implementation remains uneven across jurisdictions (Ahmed 2020; Moen 2008). As a result, balancing economic viability, environmental protection, and occupational safety continues to be one of the central challenges shaping the global ship recycling sector (Glinski 2022; Khan et al. 2012; Schulz et al. 2025).

### Efforts to improve infrastructure and management systems

In recent years, several SRFs in India and Bangladesh have undertaken substantial investments to upgrade infrastructure, management systems, and certification processes, reflecting efforts to improve alignment with international standards (Mehtaj et al. 2024). These initiatives include the construction of impermeable cutting surfaces, the installation of controlled drainage and containment systems, the implementation of formal hazardous-waste management plans, and the adoption of mechanized handling equipment aimed at reducing occupational risks (Dipto et al. 2023).

Parallel to these infrastructural upgrades, several SRFs have sought internationally recognized certifications, such as ISO14001, ISO 9001, and ISO 45001, and have obtained Statements of Compliance issued by classification societies, including DNV, ClassNK, and RINA, in relation to the requirements of the Hong Kong Convention (Gunbeyaz et al. 2019). In India, particularly in Alang, these developments have included the modernization of dismantling areas, with paved working surfaces and hazardous-waste treatment facilities. Similarly, in Bangladesh, some yards have begun replacing manual cutting operations with mechanized processes, representing incremental efforts toward safer and more controlled working conditions (Schulz et al. 2025).

Although these developments demonstrate ongoing efforts to improve ship recycling practices, their broader implications for the global ship recycling market remain uncertain. On the one hand, the upgrades may enhance the international competitiveness of certain SRFs by offering safer operational environments while maintaining comparatively lower labour and infrastructure costs. On the other hand, gaps remain in relation to several requirements of EU SRR No. 1257/2013, particularly those concerning containment, traceability, and governance requirements applicable to the EU List assessment process. These remaining gaps are observed within a broader context of structural and economic constraints that may pose challenges to achieving full compliance.

Taken together, these investments suggest a gradual trend toward greater alignment with international regulatory requirements, indicating that some SRFs in South Asia may, in the long term, move closer to meeting the operational and environmental standards established under the EU regulatory framework. However, structural challenges, including limited capital availability, governance limitations, and infrastructural deficits, may continue to pose challenges to achieving full alignment (Dipto et al. 2023).

## Methodology

Qualitative research methods are widely used to generate in-depth understanding of complex phenomena, particularly when processes, decisions, and interactions require contextual interpretation (Lim 2024). Qualitative designs may be guided by a conceptual framework, suggesting a predominantly deductive orientation, or may be driven by patterns identified within the data (Mihas 2023). Such approaches are particularly useful in naturalistic settings where human interactions, institutional behaviours, and contextual dynamics are relevant to the interpretation of observed phenomena (Delgado Hito and Romero García, 2021).

In this study, an archival research strategy was employed for qualitative data collection. Archival methods encompass a wide range of pre-existing documents and records, which can serve as valuable sources of evidence for researchers examining formal procedures, institutional practices, and regulatory processes (Lim 2024). The use of archival document analysis was considered appropriate given the nature of the phenomenon under investigation, an international regulatory process based on technical audits, and the need to examine formal evidence produced by independent classification societies. These sources support the identification of patterns associated with different EU List outcomes, the examination of criteria documented in the evaluations, and the interpretation of differences observed across the assessed facilities, which are relevant to addressing the research question.

### Data collection

Data were collected on 01 July 2025 from official inspection reports published by the European Commission. The final dataset consisted of 22 inspection reports, corresponding to 22 SRFs, each documenting technical, environmental, and operational requirements considered in the assessment of SRFs located outside the European Union. These reports were prepared by DNV as part of on-site assessments conducted on behalf of the European Commission under EU SRR No. 1257/2013. Although the analysis relied on inspection reports prepared by a single classification society, the comparative approach focused on cross-case patterns and recurring assessment criteria, while the potential limitations associated with a single-source dataset were acknowledged.

During the data screening process, an inconsistency was identified in the document Final Report Second Inspection Request 003 V2. The downloaded file did not correspond to the Turkish facility LEYAL DEMTAŞ GEMI SÖKÜM SANAYİ VE TİCARET A.Ş. but instead referred to an Indian SRF. As the correct version of the document could not be retrieved from the available source, the corresponding facility was excluded from the comparative analysis, and this issue was acknowledged as a methodological limitation. This decision was made to avoid introducing information that did not correspond to the facility under assessment into the dataset.

### Analytical categorization

Based on the structure and content of the inspection reports, analytical categories and subcategories were developed to facilitate a systematic comparison of the assessed SRFs, as presented in Table 1. The EU List status of each facility (listed or non-listed) was also recorded and used as a grouping variable in the comparative analysis.

**Table 1:** Categories and subcategories identified

Category	Category code	Subcategory	Subcategory code
Legal Authorization and Regulatory Compliance	LAC	- Approval by competent authorities - Environmental license - Operational license	- ACA - EL - OL
Project Design and Infrastructure	PI	- Dismantling method - Presence of impermeable flooring with drainage systems - Primary and secondary cutting areas - Waste storage facilities	- DM - PIF - PSC - WSF
Management and Organizational Structure	MOS	- Recycling facility plan - Functional organizational chart and allocation of responsibilities - ISO certifications	- RFP - FOC - IC
Health, Safety and Environmental Performance	HSE	- Emergency response plan - Personal protective equipment - Soil environmental monitoring systems - Water environmental monitoring systems - Air environmental monitoring systems - Noise safety monitoring systems - Intertidal zone control - Regular medical surveillance - Training monitoring and documentation	- ERP - PPE - SMS - WMS - AMS - NSM - IZC - RMS - TMD
Recycling Operations	RO	- Ship reception process - Ship Recycling Plan (SRP) - Cutting and safety procedures - Access and movement control	- SRS - SRP - CSP - AMC
Hazardous Waste and Materials Management	HWM	- Treatment of hazardous waste - Treatment of non-hazardous waste - Documentation for waste traceability - Temporary storage - Transport oversight and tracking	- THW - TNW - DWT - TS - TOT
Indicators and Monitoring	IM	- Record of accidents, incidents, and occupational diseases - Progress reports (internal or client-mandated) - Performance indicators (KPIs)	- AID - PR - PI

The development of these categories and subcategories followed a three-step methodological procedure combining deductive and inductive approaches. First, an initial deductive framework was established based on the requirements of the EU SRR No. 1257/2013, the corresponding commission implementation decisions issued between 2016 and 2024, and relevant technical guidelines published by classification societies. Second, an inductive refinement stage was conducted through exploratory reading of the inspection reports, through which additional subcategories were identified where operational, environmental, management-related, or regulatory aspects were not fully captured by the initial framework. Finally, the analytical framework underwent an iterative review and recoding process to improve conceptual clarity, reduce redundancies, and maintain consistency with the study objectives.

### Comparative analysis and parameter

A comparative assessment of listed and non-listed SRFs was conducted across the predefined analytical categories. The assessment was carried out by the authors based on the inspection reports available for each facility, applying the same final set of analytical parameters across all cases. The classifications were reviewed and discussed by the researchers to promote consistency in the interpretation of the documentary evidence.

The “not validated” (NV) classification was assigned by the researchers when the information required to assess a specific subcategory was absent, insufficient, unclear, or could not be reliably verified in the publicly available facility report. Each report was systematically reviewed, and whenever sufficient evidence was available, the corresponding subcategory assessment was classified as compliant (C), non-compliant (NC), or partially compliant (PC). NV therefore represents insufficient evidence for verification rather than a confirmed compliance status and should not be interpreted as equivalent to NC. In the context of the EU List assessment process, in which documentary evidence is used to verify compliance with applicable requirements, such documentary gaps were considered relevant to the comparative analysis.

For this reason, NV was retained as a separate classification in the quantitative assessment and included in the overall distribution of classifications, so that C, PC, NC, and NV classifications collectively represent 100% of the subcategory-level observations. NV percentages were therefore interpreted as reflecting limitations in the completeness and verifiability of the available evidence rather than confirmed operational non-compliance.

For each analytical category and facility group, the absolute frequencies of compliant (C), non-compliant (NC), partially compliant (PC), and not validated (NV) classifications were aggregated across the corresponding subcategories. For each subcategory, NI represents the number of facilities evaluated, while ΣNI represents the sum of the facilities evaluated across all subcategories within the corresponding analytical category and facility group. Thus, ΣNI corresponds to the total number of subcategory-level observations included in the calculation. Percentage values were subsequently calculated as the proportion of each classification relative to this total. The formulas used for these calculations are presented in Eqs. 1–4.

1 $$\%(C)=(\Sigma C/\Sigma NI)\times 100$$

2 $$\%(NC)=(\Sigma NC/\Sigma NI)\times 100$$

3 $$\%(PC)=(\Sigma PC/\Sigma NI)\times 100$$

4 $$\%(NV)=(\Sigma NV/\Sigma NI)\times 100$$

where ΣC, ΣNC, ΣPC, and ΣNV represent the total frequencies of each classification across the subcategories, and ΣNI represents the total number of subcategory-level observations within the corresponding analytical category and facility group. Accordingly, C, NC, PC, and NV collectively account for 100% of the observations included in each calculation.

The comparative analysis was used to examine patterns in the distribution of classifications across listed and non-listed SRFs and to identify the analytical dimensions in which differences between the two groups were observed. To support this comparison, a heat map was developed to visually represent differences in classification patterns between listed and non-listed facilities, facilitating the interpretation of variations in compliance across the assessed dimensions.

### Research limitations

This study is subject to limitations associated with its exclusive reliance on publicly available documents. These include (i) the lack of access to non-public documentation from the European Commission; (ii) gaps or redacted information in reports concerning non-listed SRFs; (iii) variations in structure and level of detail across documents; and (iv) occasional inconsistencies, such as the previously described document inconsistency. Nevertheless, the available documentation provided a consistent basis for examining recurring patterns across the assessed listed and non-listed facilities.

### Sensitivity analysis excluding facilities employing dismantling methods incompatible with EU SRR requirements

To examine whether the observed differences between listed and non-listed facilities were related primarily to dismantling-method restrictions or remained evident across other analytical dimensions, an additional sensitivity analysis was conducted by excluding SRFs employing beaching or intertidal dismantling methods considered incompatible with the applicable technical and environmental requirements of EU SRR No. 1257/2013. Following the exclusion of these facilities, the comparative assessment was recalculated across all analytical categories to examine whether the previously observed differences in classification patterns remained evident. The objective of this procedure was to evaluate the robustness of the analytical interpretation and assess whether differences between listed and non-listed facilities remained evident in governance-related dimensions, particularly environmental monitoring, traceability systems, and institutional management structures, after facilities subject to dismantling-method restrictions were excluded. The results of this complementary analysis were subsequently compared with those obtained from the original dataset to examine the extent to which the observed patterns changed following the exclusion of these facilities.

## Results

Among the 22 SRFs included in the comparative assessment, 12 (54.5%) were included in the European List and 10 (45.5%) were non-listed, as presented in Table 2. Variations in the distribution of C, PC, NC, and NV classifications were observed across the analytical categories and between the two groups.

**Table 2:** SRFs that applied for inclusion in the EU List during the period 2018 to 2024

Facilities included in the EU List				Facilities not included in the EU List			
Application number	Facilities name	Country	Recycling method	Application number	Facilities name	Country	Recycling method
04	International Shipbreaking Limited, L.L.C. Facility	USA	Floating dock with ramp	03	Priya Blue Industries Pvt Ltd	India	Intertidal stranding/Landing
12	Avsar Gemi Sokum San Dis. Tic. Ltd. Sti	Turkey	Launch ramp landing	06	Shree Ram Vessel scrap Pvt. Ltd	India	Intertidal stranding/Landing
13	Öge Gemi Söküm Ithalat Ihracat Ticaret Ve Sanayi AŞ	Turkey	Slipway landing method	07	Leela Ship Recycling Private Limited	India	Stranding/Landing between tides
15	LEYAL GEMİ SÖKÜM SANAYÎ ve TÍCARET LTD	Turkey	Combination of floating dismantling and ramp	23	Y. S Investments	India	Intertidal landing
26	EGE CELIK SAN. VE TIC. A.S	Turkey	Floating dismantling and landing	24	R. K. Industries (Unit-II) LLP	India	Intertidal grounding/Landing
32	Temurtaslar Gemi Söküm	Turkey	Ramp landing	27	J.R.D. Industries	India	Beaching/Intertidal landing method
34	Ege Gemi Söküm San. ve. Tic. A.S	Turkey	Slipway landing	40	Dortel Gemi Sokum Demir Celik Sanayi Ve Tic.Ltd. Sti	Turkey	Unloading via ramp combining floating dismantling with land-based dismantling
36	Anadolu Gemi Söküm Orman Ürn. Gida Tur. Nak, San. Ve Tic. A.Ş	Turkey	Combination of floating and land-based dismantling and ramp beaching	49	Kiliçlar Geri Dönüşümlü Maddeler Ve Metal San.Tic A.Ş	Turkey	Beaching
38	BMS Gemi Geri Donusum San ve Tic. A.S	Turkey	Slipway landing method	51	Blade Denizcilik Geri Dönüşüm San. Ve Tic. A.Ş	Turkey	Beaching
42	Dales Marine Services Ltd	UK	Dry dock	56	Işiksan Ship Recycling	Turkey	Landing method
50	Kishorn Port Limited	UK	Dry dock				
14	Sök Denizcilik Tic.Ltd.Sti	Turkey	Floating dismantling and ramp landing				

**Fig. 1:**
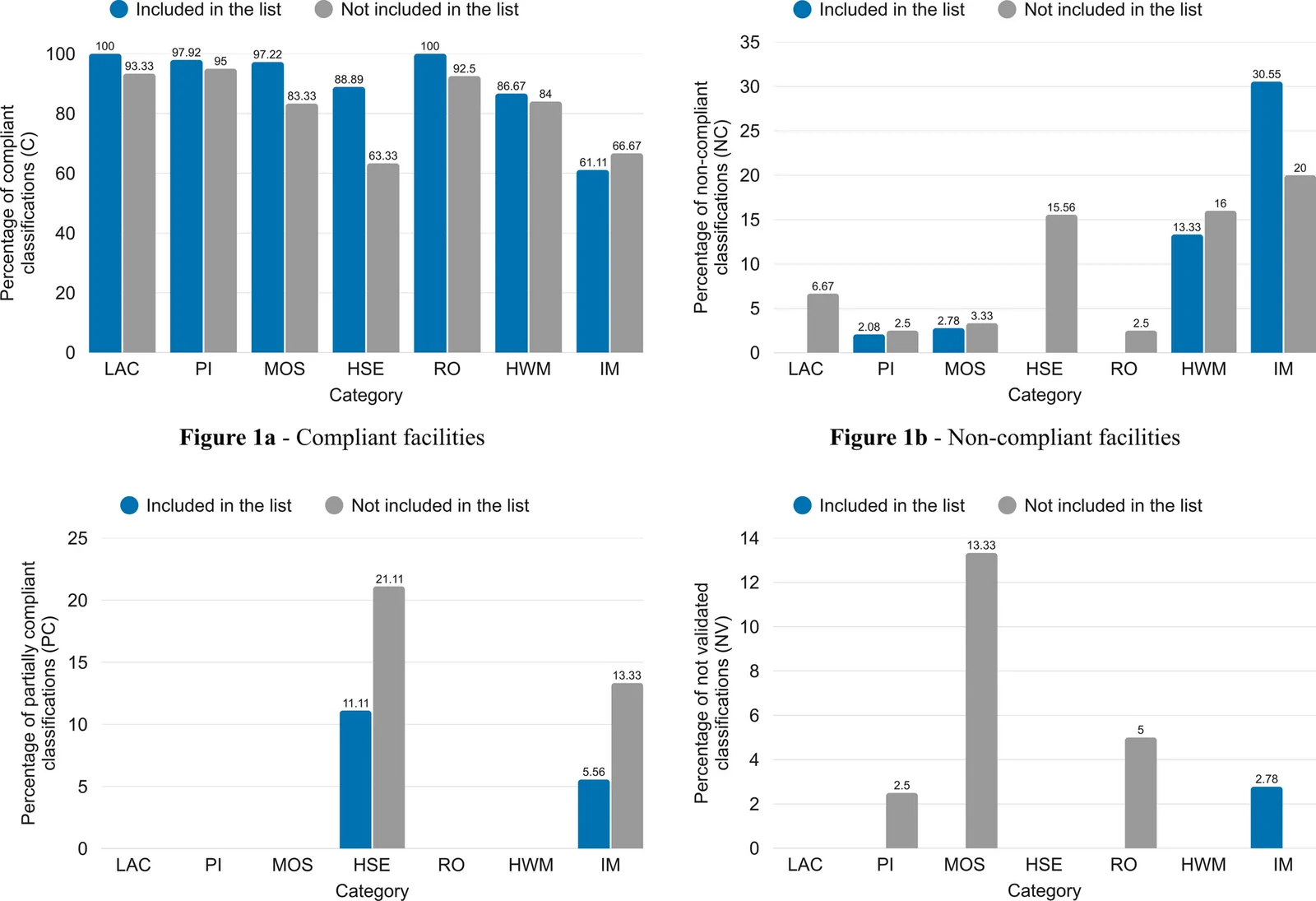
Parameters analyses

Figure 1 summarizes the distribution of classifications across the two groups of SRFs. Considering all analytical categories, compliant classifications accounted for 90.26% of the aggregated subcategory-level observations among listed facilities and 82.59% among non-listed facilities. Examination at the category level showed that the magnitude of the differences between listed and non-listed facilities varied across the assessed dimensions.

Geographical differences were also observed in the distribution of the assessed facilities. Listed SRFs were predominantly located in Turkey, the United Kingdom (UK), and the United States of America (USA), whereas non-listed SRFs were concentrated mainly in India and Turkey. Across the listed facilities, the inspection reports documented varying degrees of mechanization, paved and controlled working areas, and ISO certifications. Among non-listed facilities, beaching-based dismantling methods were more frequently documented, while differences were also observed in environmental monitoring, HSE practices, and performance indicators.

Figure 1 presents the distribution of classifications across the analytical categories for listed and non-listed facilities. Figure 1a presents the proportions of C classifications, while Fig. 1b, c, and d present the corresponding proportions of NC, PC, and NV classifications, respectively. In all panels, the blue bars represent the classification proportions for listed facilities, whereas the grey bars represent those for non-listed facilities. The category-level comparison showed that the magnitude and distribution of differences varied across the assessed dimensions. Non-listed facilities show higher percentages of NC, PC, or NV classifications in specific categories, particularly Health, Safety and Environmental Performance, Indicators and Monitoring, and Hazardous Waste and Materials Management. NV classifications, however, should be interpreted as limitations in the available documentary evidence rather than as confirmed non-compliance.

**Fig. 2:**
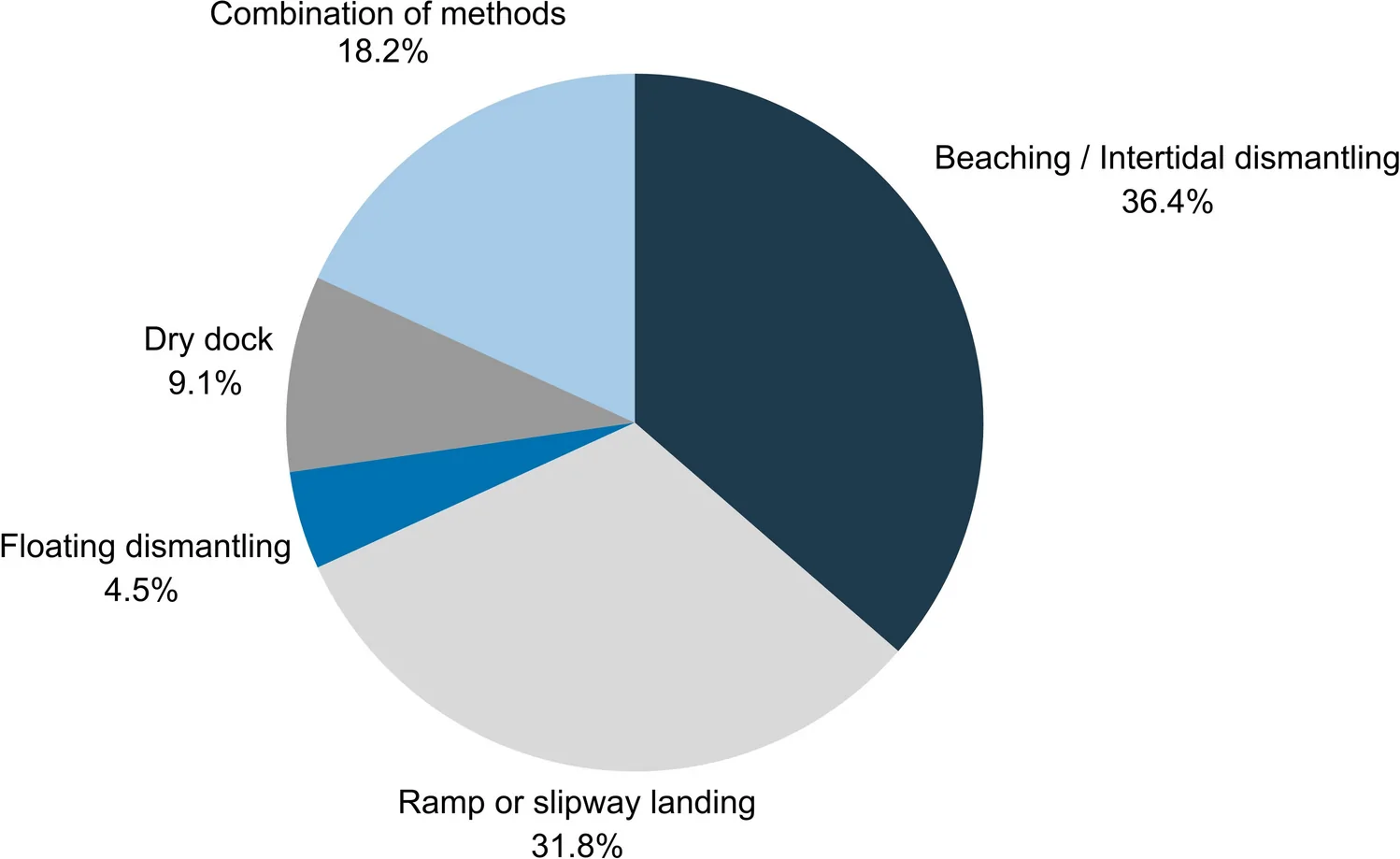
Facilities recycling method

Figure 2 presents the distribution of dismantling methods documented among the assessed SRFs. Beaching/intertidal dismantling was the most frequently documented approach (36.4%), followed by ramp or slipway landing (31.8%). Combined methods accounted for 18.2% of the assessed facilities, while dry-dock and floating dismantling represented 9.1% and 4.5%, respectively.

Comparison by EU List status showed differences in the distribution of dismantling methods among the assessed facilities (Table 2 and Fig. 2). Among the 12 listed facilities, ramp/slipway-based, floating, and combined dismantling approaches were more frequently documented, while dry-dock dismantling was documented for both assessed UK facilities. Among the 10 non-listed facilities, beaching or intertidal dismantling was more frequently documented, particularly in India, where all six assessed facilities employed some form of intertidal landing, stranding, grounding, or beaching. A similar pattern was observed among the assessed Turkish facilities: listed Turkish facilities primarily employed ramp/slipway or combined floating and land-based approaches, whereas beaching was documented for two of the four non-listed Turkish facilities.

These findings suggest that infrastructure-supported dismantling methods were more frequently documented among the listed facilities assessed in this study. Nevertheless, the observed differences should not be interpreted as evidence that dismantling method alone explains the EU List status of the assessed facilities, as the EU assessment framework encompasses multiple technical, environmental, safety, and operational requirements. Dry-dock, floating, and infrastructure-supported land-based operations may provide greater potential for the containment of wastes and effluents and for the management of occupational safety risks, although the higher capital requirements associated with these approaches may limit their adoption in some ship-recycling contexts (Devault et al. 2017). These operational characteristics are relevant to the EU regulatory framework, which establishes requirements concerning effluent containment, impermeable working surfaces, hazardous-material segregation and management, and adequate mechanical infrastructure (European Union, 2023).

### Legal Authorization and Regulatory Compliance (LAC)

The LAC category comprises three subcategories: approval by competent authorities; environmental licensing; and operational licensing. The LAC category showed high proportions of C classifications in both groups (Fig. 1). As shown in Fig. 1a, C classifications accounted for 100% of the aggregated subcategory-level observations among listed facilities and 93.33% among non-listed facilities. The high proportion of C classifications observed in both groups suggests that LAC requirements, when considered in isolation, showed limited variation between listed and non-listed facilities in the assessed sample.

The exception was Application 51, corresponding to a non-listed facility in Turkey. Its inspection report documented deficiencies in the environmental management system, including non-compliance of the facility’s asbestos-removal practices with applicable Turkish legislation. Although the facility held waste-management-related licenses, the report indicated that it did not possess a valid environmental 

### Project Design and Infrastructure (PI)

The PI category comprised the following subcategories: dismantling method; presence of impermeable flooring with drainage systems; availability of primary and secondary cutting areas; and waste storage facilities. Detailed information on the dismantling method employed by each facility was recorded, whereas the remaining subcategories were assessed based on the documented presence or absence of the corresponding infrastructure, with NV assigned when the available documentary evidence was insufficient for verification. As shown in Fig. 1a, C classifications accounted for 97.92% of the aggregated PI subcategory-level observations among listed facilities and 95.00% among non-listed facilities, indicating only a small difference between the two groups in this category.

Among the listed facilities, the principal exception was Application 42 (UK), for which the requirement related to on-site waste storage facilities was classified as non-compliant. The facility did not maintain an on-site waste storage area and instead relied on third-party companies for daily waste collection. Although waste was collected through this external arrangement, the absence of dedicated on-site waste storage infrastructure resulted in an NC classification for this subcategory.

Among the non-listed SRFs, infrastructure-related deficiencies were documented in Applications 07 and 51. Application 07 (India) lacked designated primary and secondary cutting areas, as well as impermeable flooring equipped with an appropriate drainage system. The inspection report documented heterogeneous surface conditions, with some areas paved and others only partially sealed, as well as non-standardized cutting areas, including an area referred to as the “oil zone” in the report. Similarly, Application 51 (Turkey) did not have impermeable flooring or a functional drainage system.

Overall, NC classifications represented only a small proportion of the aggregated PI subcategory-level observations and were primarily related to deficiencies in designated cutting areas and impermeable flooring with drainage systems, both of which are relevant to spill prevention and environmental control.

### Management and Organizational Structure (MOS)

This category encompasses three subcategories: recycling facility plan; functional organizational chart and allocation of responsibilities; and ISO certifications. In this category, differences in the distribution of classifications between listed and non-listed facilities were more apparent (Fig. 1). The aggregated MOS subcategory classifications showed a compliant classification rate of 97.22% for listed facilities and 83.33% for non-listed facilities, while 13.33% of the classifications for non-listed facilities were categorized as NV (Fig. 1a and d).

The recurring shortcomings observed in non-listed facilities relate to the absence, incomplete availability, or limited verifiability of the recycling facility plan, shortcomings related to the functional organizational chart and allocation of responsibilities, and insufficient evidence to verify the reported ISO certifications, such as ISO 9001, ISO 14001, ISO 45001, and ISO 30000.

Several cases exemplify these issues. The SRF evaluated in Application 07 (India) presented partially redacted documentation, insufficient evidence to verify the organizational structure, and insufficient evidence to verify ISO certifications. In the case of two Turkish facilities (Applications 49 and 51), different classification outcomes were observed regarding formal certification. For Application 49, the available documentation was insufficient to verify formal certification, resulting in an NV classification. In contrast, Application 51 was classified as NC because the documented evidence indicated that the required formal certification was not in place.

### Health, Safety and Environmental Performance (HSE)

The HSE category comprised the following subcategories: emergency response plan; personal protective equipment; soil environmental monitoring systems; water environmental monitoring systems; air environmental monitoring systems; noise safety monitoring systems; intertidal zone control; regular medical surveillance; and training monitoring and documentation. As shown in Fig. 1, C classifications accounted for 88.89% of the aggregated HSE subcategory-level observations among listed facilities and 63.33% among non-listed facilities. PC classifications accounted for 11.11% and 21.11%, respectively, while NC classifications were not observed among listed facilities but accounted for 15.56% of the observations among non-listed facilities.

A facility-level examination of the underlying classifications showed that five listed SRFs received C classifications across all assessed HSE subcategories (Applications 26, 32, 34, 42, and 14). The remaining seven listed facilities presented at least one PC classification, particularly in relation to soil environmental monitoring. Among the non-listed SRFs, only Applications 40 and 56 received C classifications across all assessed HSE subcategories, whereas the remaining eight presented one or more NC, PC, or NV classifications. Application 07 illustrates the different classifications observed across the HSE subcategories. Soil and noise monitoring, as well as intertidal zone control, were classified as non-compliant, while air monitoring and regular medical surveillance were classified as partially compliant. In contrast, the emergency response plan, personal protective equipment, water monitoring, and training monitoring and documentation were classified as compliant.

### Recycling Operations (RO)

The RO category comprised four subcategories: ship reception process; Ship Recycling Plan (SRP); cutting and safety procedures; and access and movement control. Based on the inspection reports, the ship reception process was generally organized into two main stages. The first stage, preparation and pre-arrival documentation, involved contract analysis, verification of acceptance criteria, review of the Inventory of Hazardous Materials (IHM), preparation or updating of the SRP, verification of flag documentation, and pre-cleaning procedures. The second stage, arrival and securing of the vessel, included safe berthing, initial safety inspections, and authorization to initiate dismantling activities.

As shown in Fig. 1, C classifications accounted for 100% of the aggregated RO subcategory-level observations among listed facilities and 92.5% among non-listed facilities. Among non-listed facilities, NC and NV classifications accounted for 2.5% and 5.0% of the aggregated RO subcategory-level observations, respectively. At the facility level, the ship reception process received a C classification for all assessed SRFs, irrespective of EU List status.

Greater variation was observed in the access and movement control subcategory. Some SRFs employed access-control systems that included continuous surveillance, cameras, identification cards, and, in some cases, QR-code-based management systems. Other facilities relied on simpler access-control procedures, such as manual sign-in sheets. At the Turkish facility Dortel Gemi Sokum, the inspection report considered this procedure insufficient because of its susceptibility to manipulation and limited traceability.

### Hazardous Waste and Materials Management (HWM)

The HWM category comprised the following subcategories: treatment of hazardous waste; treatment of non-hazardous waste; documentation for waste traceability; temporary storage; and transport oversight and tracking. As shown in Fig. 1, C classifications accounted for 86.67% and 84.00% of the aggregated HWM subcategory-level observations among listed and non-listed facilities, respectively, while NC classifications accounted for 13.33% and 16.00%.

The qualitative examination of the inspection reports provided additional context for these classification patterns. Among listed SRFs, the final treatment of hazardous and non-hazardous waste was frequently outsourced to third-party operators, while on-site waste-management activities were primarily related to temporary storage and material segregation.

Outsourced waste treatment was documented among both listed and non-listed facilities and was therefore not restricted to either group in the assessed sample. Differences were instead observed in the documentation and verifiability of waste flows, destinations, traceability mechanisms, and arrangements with external operators. Among non-listed facilities, documented shortcomings were more frequently related to incomplete waste-flow records and limited evidence regarding waste destinations and subsequent treatment arrangements.

### Indicators and Monitoring (IM)

The IM category comprised three subcategories: record of accidents, incidents, and occupational diseases; progress reports (internal or client-mandated); and performance indicators (KPIs). As shown in Fig. 1, the IM category exhibited a mixed distribution of C, NC, PC, and NV classifications across listed and non-listed facilities. C classifications accounted for 61.11% of the aggregated IM subcategory-level observations among listed facilities and 66.67% among non-listed facilities. NC classifications accounted for 30.56% and 20.00%, respectively, while PC classifications represented 5.56% and 13.33%. NV classifications accounted for 2.78% of the observations among listed facilities, whereas no NV classifications were recorded among non-listed facilities. Unlike the patterns observed in some other analytical categories, the aggregated IM results did not show a consistently higher proportion of C classifications or a lower proportion of NC classifications among listed facilities.

At the facility level, several listed SRFs did not present fully structured KPI systems or formalized progress-reporting mechanisms, with some inspection reports documenting practices such as near-miss reporting and brief safety meetings rather than comprehensive KPI and progress-reporting systems. In other cases, safety and performance management frameworks were documented, but the available evidence lacked sufficient standardization or detail to support verification of the corresponding parameters.

Among non-listed SRFs, some monitoring systems were reported as being under implementation, with the inspection reports documenting limitations related to the definition of objectives, assignment of responsibilities, and integration of monitoring information into operational processes. These facility-level observations are consistent with the mixed aggregated pattern shown in Fig. 1, in which both listed and non-listed facilities presented limitations across different IM subcategories. Overall, the IM category did not show a consistent pattern of more favourable classifications among listed facilities. Instead, the observed results reflected variation in the degree of formalization and documentation of monitoring and performance-management practices across the assessed facilities. More broadly, the direction and magnitude of the differences between listed and non-listed facilities varied across the analytical categories, with more pronounced differences observed in dimensions such as HSE and MOS.

### Heat map

**Fig. 3:**
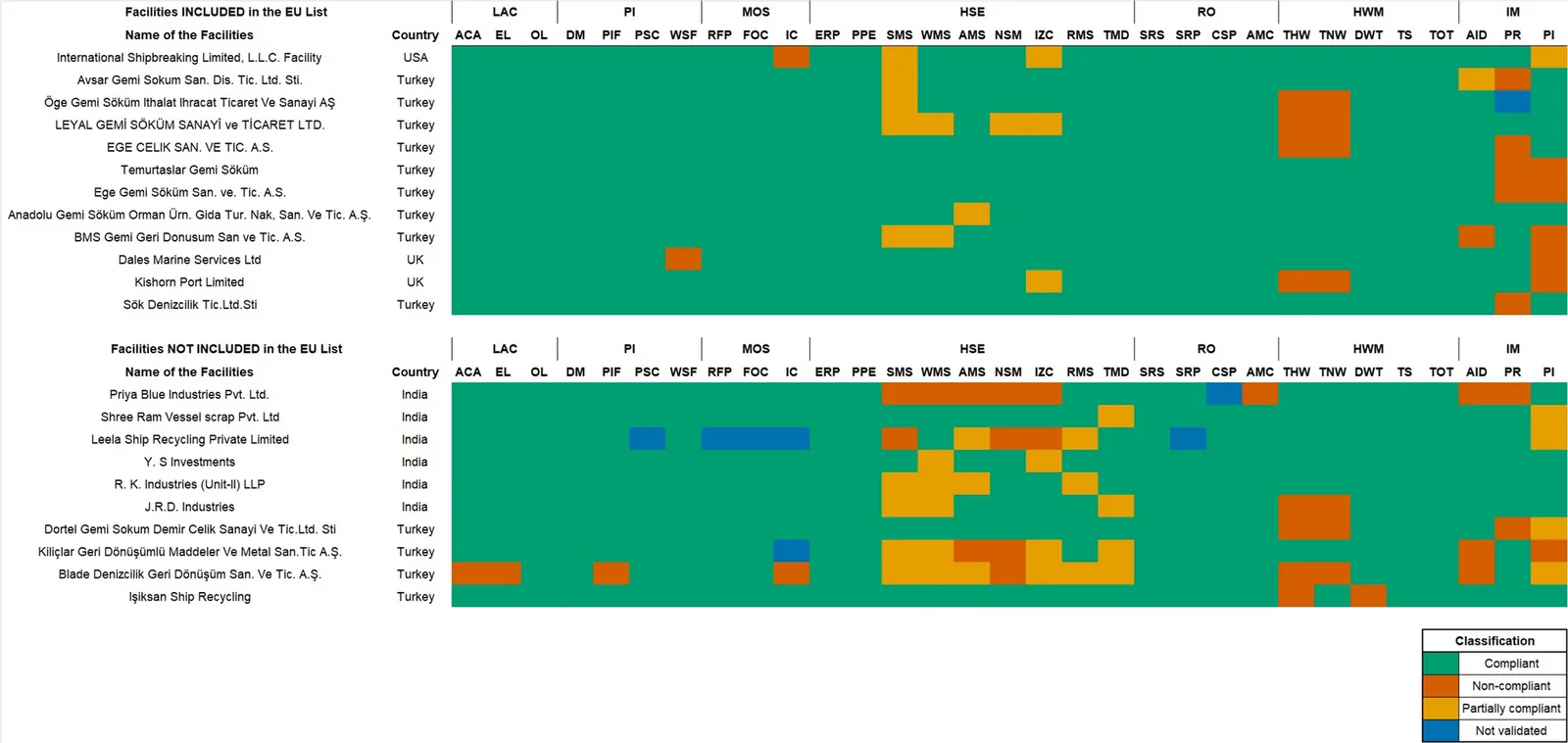
Heat map of the SRFs assessed for inclusion in the EU List

A heat map was developed to visually summarize the classification patterns across the assessed SRFs (Fig. 3). The figure shows the distribution of C, PC, NC, and NV classifications across the analytical categories (LAC, PI, MOS, HSE, RO, HWM, and IM), allowing visual comparison of patterns across listed and non-listed facilities. The classification patterns shown in Fig. 3 were not restricted to technical and infrastructure-related dimensions but were also observed across governance, environmental monitoring, documentation, and control-related subcategories. However, the direction and magnitude of these differences varied across categories, and limitations in IM-related parameters were observed in both listed and non-listed facilities. Accordingly, the heat map should be interpreted as a visual synthesis of the classification patterns documented across the assessed SRFs rather than as evidence that any individual criterion independently explains EU List status.

The heat map analysis (Fig. 3) shows differences in the distribution of conformities and non-conformities between listed and non-listed facilities. Although each SRF is assessed individually, geographical differences in the distribution of conformities and non-conformities are apparent across the assessed facilities, suggesting potential variation across national and institutional contexts.

Notably, deficiencies in IM are distributed across both listed and non-listed facilities, suggesting that this category does not show a consistent pattern of differentiation between listed and non-listed facilities when considered in isolation. Instead, IM-related deficiencies are also observed among listed facilities that demonstrate stronger performance in other categories such as environmental governance, traceability systems, and operational risk control. This pattern suggests that IM should be interpreted in conjunction with other dimensions rather than as an isolated indicator of differences between the two groups.

The distribution of non-conformities across the heat map indicates that some categories differentiate the two groups. Persistent deficiencies in environmental monitoring, intertidal zone control, and governance systems were more frequently observed among non-listed facilities, whereas isolated shortcomings in KPI systems or reporting structures were observed in both groups and therefore did not consistently differentiate listed from non-listed facilities. These results suggest that the magnitude and consistency of the observed differences vary across analytical categories.

The results show that facilities included in the EU List did not necessarily exhibit complete conformity across all assessed dimensions. Rather, listed facilities could present deficiencies in some dimensions while maintaining stronger evidence of continuous management and verification of operational and environmental risks.

Within the assessed sample, non-listed SRFs were primarily located in India and Turkey and exhibited non-conformities across several assessed dimensions, including HSE, HWM, and IM (Hsuan and Parisi 2020; Ignacio Alcaide et al. 2017). These facilities were frequently classified as “NV”, reflecting documentary gaps or insufficient verifiable evidence in the available reports.

These limitations were observed not only in relation to physical infrastructure but also in environmental governance, traceability mechanisms, and risk management practices, consistent with previous findings (Patra et al. 2023; Tola et al. 2024). However, gaps remained in several areas relevant to EU assessment requirements.

In the specific case of the assessed Indian facilities, recent improvements were observed, including the installation of impermeable flooring, expansion of cutting areas, and engagement of accredited laboratories (Devaux and Nicolaï, 2020; Ignacio Alcaide et al. 2017). Significant gaps persist in environmental monitoring, training management, operational documentation, and institutional capacity to integrate indicators into decision-making. Overall, the inspection reports documented a pattern of non-conformities, inconsistencies, and insufficient verifiable evidence across these areas.

In contrast, Turkish facilities included in the European List exhibited a different performance profile. Despite residual weaknesses, particularly in environmental aspects associated with slipway dismantling methods, these SRFs showed more established organizational structures, ISO certifications, timely responses to inspection observations, comprehensive recycling plans, and well-established administrative systems. Several facilities implemented substantial improvements between the first and second inspections, indicating responsiveness to inspection observations (Ignacio Alcaide et al. 2017).

The assessed SRFs located in the UK and USA exhibited high levels of technical and institutional robustness. Although occasional shortcomings in monitoring indicators were observed, these facilities were characterized by consolidated infrastructure, established safety mechanisms, reliable traceability systems, and consistent evidence of legal compliance. Taken together, despite a high compliance rate of 86.4% across the aggregated subcategory classifications, the results show that differences between listed and non-listed facilities are not uniformly distributed across the analytical categories. Non-conformities are concentrated in a small group of categories (HSE, HWM, and IM), although their frequency and capacity to differentiate listed and non-listed facilities vary across these categories.

The integrated analysis indicates that differences between listed and non-listed SRFs are not fully captured by average performance and are also reflected in institutional systems related to traceability, pollution control, and continuous, verifiable risk management. In summary, while infrastructure-related differences remain relevant to the assessment, environmental governance is also among the dimensions in which differences between listed and non-listed facilities were observed.

### Sensitivity analysis excluding structurally prohibited dismantling methods

A sensitivity analysis was conducted by excluding facilities employing dismantling methods prohibited under EU SRR No. 1257/2013. The results showed that some of the differences initially observed between listed and non-listed facilities were reduced after these facilities were excluded from the analysis. Notably, Applications 40 and 56, which were not included in the European List, exhibited compliance profiles broadly comparable to those of several listed SRFs across the remaining assessed dimensions once the dismantling-method criterion was excluded from the analysis. These results suggest that the observed differences between listed and non-listed facilities cannot be interpreted solely based on cumulative compliance performance or governance-related dimensions.

After the exclusion of facilities employing structurally prohibited dismantling methods, differences in environmental monitoring, traceability, governance, and risk-control systems nevertheless remained evident between the two groups. Thus, the sensitivity analysis indicates that the observed assessment patterns encompass both dismantling-method requirements and differences across governance-related dimensions, without implying that any single dimension independently determines listing outcomes.

## Discussion

The results suggest that EU List outcomes reflect different patterns of conformity and non-conformity across the assessed dimensions, with persistent failures in environmental monitoring and governance more frequently observed among non-listed facilities, despite relatively high overall compliance levels. This research indicates that differences between listed and non-listed SRFs are not fully captured by the total number of fulfilled requirements but are also reflected in the distribution of non-conformities, particularly within the categories of governance, environmental monitoring, and safety systems. This conclusion aligns with previous research showing that international regulatory frameworks, such as the EU SRR No. 1257/2013, assess not only the physical infrastructure of facilities but also their institutional capacity to manage risks in a systematic, documented, and verifiable manner (European Union, 2023).

The analysis further suggests that environmental governance is one of the dimensions associated with differences between listing outcomes. The assessed SRFs located in India and Turkey exhibited notable documentary deficiencies, including the absence of functional organizational charts, incomplete ISO certifications, and inconsistencies in Ship Recycling Facility Plans (SRFPs). The literature emphasizes that institutional maturity is essential for ensuring occupational safety and full traceability of hazardous waste management (Misra 2019; Rahman et al. 2018).

This research also raises important questions regarding the growing dependence of contemporary regulatory systems on documentary evidence as a proxy for operational safety and environmental performance. Although listed facilities generally demonstrated stronger governance structures, certification systems, and traceability mechanisms, the results do not necessarily imply that documented compliance fully reflects the functioning of industrial operations. As argued by Dekker (2014) in Bureaucratization of Safety, complex management systems may progressively prioritize the production of auditable documentation over the effective management of operational risks, creating a potential gap between formal compliance and practical safety performance. In this respect, the stronger performance observed among facilities located in the UK and USA may partially reflect their longer-standing institutional experience in managing complex bureaucratic and certification systems rather than exclusively superior operational conditions. This finding suggests that the current regulatory trend toward increasingly documentation-intensive auditing frameworks should be critically examined, particularly in highly dynamic industrial settings such as ship recycling, where operational realities may not always be fully captured through formal records alone.

Nevertheless, the study does not suggest that documentation systems are unnecessary. Rather, the findings indicate that documentary compliance should be interpreted as one component of a broader institutional capability framework that must also include operational verification, environmental performance consistency, and adaptive risk management capacity.

A second key finding concerns environmental monitoring, where differences between listed and non-listed facilities were observed. Widespread failures in soil, water, air, and noise monitoring were observed among non-listed SRFs. This pattern is consistent with studies showing that even when structural improvements are implemented in Alang and Chittagong, the monitoring of critical environmental parameters remains incomplete or inconsistent (Hasan et al. 2020; Schulz et al. 2025). In the present analysis, the absence or insufficiency of environmental data was frequently observed among non-listed facilities, including facilities with otherwise reasonable infrastructure.

Another notable finding concerns the management of the intertidal zone. Among the assessed facilities, SRFs primarily employing beaching or landing methods in tropical coastal regions exhibited higher numbers of non-conformities, particularly those related to spill control, metallic debris, and oily runoff. The environmental vulnerability of the intertidal zone is well documented in the literature, which identifies this area as particularly susceptible to severe and persistent environmental impacts (Devault et al. 2017; Dewan and Sibilia 2024). Research conducted in Bangladesh, for instance, highlights chronic contamination of sediments and coastal biota by heavy metals (Cu, Pb, Cd), organotin compounds, and microcontaminants, with impacts that are difficult to mitigate without adequate physical containment (Rahman et al. 2018; Roy et al. 2022).

Additionally, the results show that infrastructure, while relevant, does not clearly differentiate listed and non-listed facilities when considered in isolation. High levels of conformity in “Design and Infrastructure” across both groups indicate that physical improvements have become widespread, a trend already observed in recent studies on the gradual modernization of shipyards in Turkey and India (Abdullah et al. 2023; Akter and Arifuzzaman 2023). Nevertheless, the results indicate that such improvements may coexist with shortcomings in governance and in the capacity to demonstrate sustained compliance over time.

The results suggest an apparent paradox: several listed SRFs also exhibit shortcomings in IM. However, the qualitative analysis suggests that these deficiencies follow different patterns across the two groups. In listed facilities, monitoring activities tend to be varied but substantive, with records of near misses, internal audits, and corrective procedures. In non-listed facilities, by contrast, monitoring is incipient, fragmented, and disconnected from decision-making processes, a pattern consistent with literature documenting systemic monitoring deficiencies in SRFs across the Global South (Glinski 2022; Khan et al. 2012).

Thus, differences between listed and non-listed facilities are not fully explained by cumulative compliance across all evaluated categories but are also reflected in the distribution of non-conformities across specific dimensions. The heat map analysis revealed that several facilities included in the EU List still exhibited isolated non-conformities, particularly in IM, HWM, and selected HSE parameters, indicating that certain deficiencies may coexist with inclusion in the EU List, including in facilities that also present robust governance, environmental control, and traceability systems. In contrast, persistent shortcomings in environmental monitoring, intertidal zone control, operational traceability, and institutional governance were more frequently observed among non-listed facilities. These patterns highlight differences between the groups in the documented evidence of continuous management of operational and environmental conditions. Furthermore, the sensitivity analysis excluding facilities operating under structurally prohibited dismantling methods showed that differences between the two groups remained evident in governance-related dimensions, suggesting that the observed assessment patterns extend beyond differences related solely to dismantling infrastructure and encompass institutional capacity, auditability, and verifiable risk management systems.

Taken together, these findings contribute to the literature by providing empirical evidence of differences between listed and non-listed facilities in the systemic coherence of governance and environmental control mechanisms, rather than in any single parameter. While confirming earlier insights, the study contributes new evidence by showing how these differences manifest in inspection reports, thereby addressing a gap that has received limited attention in previous research.

## Conclusions

This study sought to identify patterns associated with different listing outcomes among SRFs evaluated for inclusion in the European List. The results suggest that differences between listed and non-listed facilities are not fully captured by the total number of requirements met but are also reflected in the nature and distribution of non-conformities across specific assessed dimensions. Facilities that failed to provide consistent evidence of soil, water, air, and noise monitoring, or that exhibited documentary gaps in management plans, showed limitations in demonstrating compliance with the requirements considered in the EU assessment process, despite physical improvements implemented in recent years.

The analysis also highlights the intertidal zone as one of the dimensions associated with differences in listing outcomes. Methods such as beaching, which are prevalent in India and other parts of South Asia, present challenges for effective containment and the prevention of recurrent leakage conditions that may pose challenges to meeting the environmental protection requirements established under the EU SRR. In contrast, the assessed facilities located in Turkey, the UK and USA generally showed more consistent integration of infrastructure, management practices, and monitoring systems, even when minor limitations in indicators or performance reporting persisted.

An additional contribution of this study emerges from the sensitivity analysis excluding facilities employing dismantling methods prohibited under the EU SRR No. 1257/2013. Under this scenario, Applications 40 and 56 displayed performance patterns broadly comparable to those of several listed facilities across the remaining assessed dimensions, suggesting that their non-listing outcomes cannot be interpreted solely based on overall compliance performance. This finding further indicates that structural eligibility criteria alone do not explain all observed differences between listed and non-listed facilities, as differences in governance capability, environmental monitoring, traceability, and risk-control systems remained evident between the two groups. Overall, differences between listed and non-listed facilities were observed not only in infrastructure and operational practices but also in specific dimensions related to environmentally sound ship recycling.

Thus, the observed differences between listed and non-listed SRFs are reflected not only in compliance across individual categories but also in governance, environmental evidence, and operational control. The central conclusion of this study is that environmentally sound ship recycling is associated with institutional systems capable of supporting consistency, verifiability, and continuous improvement. This perspective extends beyond physical compliance and contributes to a broader understanding of the institutional and operational characteristics associated with environmentally sound and socially responsible ship recycling. By examining differences in governance, environmental monitoring, documentation, and operational control between listed and non-listed facilities, this study contributes to a clearer understanding of the institutional dimensions of environmentally sound ship recycling and highlights the recurrent differences in governance observed between the two groups.

## Supplementary Information

Below is the link to the electronic supplementary material.

ESM 1 DOCX (56.0 KB)

## Data Availability

The authors declare that the data supporting the findings of this study are available within this paper.
