# Supplementary material for: Compliance and non-compliance patterns among listed and non-listed ship recycling facilities: a comparative assessment of 22 facilities evaluated for the EU List
Source: Environ Sci Pollut Res Int. 2026 Oct 3;33(34):16797–813. doi: 10.1007/s11356-026-38259-w (PMC13680528; doi:10.1007/s11356-026-38259-w)
Supplement: Supplementary file 1 [file 11356_2026_38259_MOESM1_ESM.docx]

**Calculation of percentages by parameter for subcategories - Facilities included in the EU List**

Definitions:

*C* = Compliant

*NC* = Non-compliant

*PC* = Partially compliant

*NV* = Not validated

*NI* = Number of installations analysed

$\%(C)=(C/NI)\times100$	(1)

$\%(NC)=(NC/NI)\times100$	(2)

$\%(PC)=(PC/NI)\times100$	(3)

$\%(NV)=(NV/NI)\times100$	(4)

Using Final Report Application 007 V4 as an example, two possible responses were adopted for the Emergency Response Plan (ERP) subcategory within the Health, Safety and Environmental Performance (HSE) category: “Yes” (Compliant) or “No” (Non-compliant). A “Yes” classification was assigned when the facility had an Emergency Response Plan in place, as was the case for this application.

For the Soil Environmental Monitoring Systems (SMS) subcategory, values of 5, 3, and 1 corresponded to Compliant, Partially Compliant, and Non-compliant classifications, respectively. For the application analysed, a value of 1 (Non-compliant) was assigned because, although “Soil” was listed as an environmental parameter to be considered or assessed in the results section of the evaluation, the corresponding compliance field was left blank.

Cases in which the available information was incomplete or insufficient to support a classification as Compliant, Partially Compliant, or Non-compliant were classified as not validated (NV). For example, in this application, within the Management and Organizational Structure (MOS) category, compliance with the Emergency Response Plan (ERP) subcategory could not be validated due to insufficient documentary evidence.

**Category: Legal Authorization and Regulatory Compliance (LAC)**

**Subcategory: Approval by competent authorities (ACA)**

Values: *C* = 12, *NC* = 0, *PC* = 0, *NV* = 0, *NI* = 12

Calculations:

$\%(C)=(12/12)\times100=100.00\%$	(1)

$\%(NC)=(0/12)\times100=0.00\%$	(2)

$\%(PC)=(0/12)\times100=0.00\%$	(3)

$\%(NV)=(0/12)\times100=0.00\%$	(4)

**Subcategory: Environmental license (EL)**

Values: *C* = 12, *NC* = 0, *PC* = 0, *NV* = 0, *NI* = 12

Calculations:

$\%(C)=(12/12)\times100=100.00\%$	(1)

$\%(NC)=(0/12)\times100=0.00\%$	(2)

$\%(PC)=(0/12)\times100=0.00\%$	(3)

$\%(NV)=(0/12)\times100=0.00\%$	(4)

**Subcategory: Operational license (OL)**

Values: *C* = 12, *NC* = 0, *PC* = 0, *NV* = 0, *NI* = 12

Calculations:

$\%(C)=(12/12)\times100=100.00\%$	(1)

$\%(NC)=(0/12)\times100=0.00\%$	(2)

$\%(PC)=(0/12)\times100=0.00\%$	(3)

$\%(NV)=(0/12)\times100=0.00\%$	(4)

**Category: Project Design and Infrastructure (PI)**

**Subcategory: Dismantling method (DM)**

Values: *C* = 12, *NC* = 0, *PC* = 0, *NV* = 0, *NI* = 12

Calculations:

$\%(C)=(12/12)\times100=100.00\%$	(1)

$\%(NC)=(0/12)\times100=0.00\%$	(2)

$\%(PC)=(0/12)\times100=0.00\%$	(3)

$\%(NV)=(0/12)\times100=0.00\%$	(4)

**Subcategory: Presence of impermeable flooring with drainage systems (PIF)**

Values: *C* = 12, *NC* = 0, *PC* = 0, *NV* = 0, *NI* = 12

Calculations:

$\%(C)=(12/12)\times100=100.00\%$	(1)

$\%(NC)=(0/12)\times100=0.00\%$	(2)

$\%(PC)=(0/12)\times100=0.00\%$	(3)

$\%(NV)=(0/12)\times100=0.00\%$	(4)

**Subcategory: Primary and secondary cutting areas (PSC)**

Values: *C* = 12, *NC* = 0, *PC* = 0, *NV* = 0, *NI* = 12

Calculations:

$\%(C)=(12/12)\times100=100.00\%$	(1)

$\%(NC)=(0/12)\times100=0.00\%$	(2)

$\%(PC)=(0/12)\times100=0.00\%$	(3)

$\%(NV)=(0/12)\times100=0.00\%$	(4)

**Subcategory: Waste storage facilities (WSF)**

Values: *C* = 11, *NC* = 1, *PC* = 0, *NV* = 0, *NI* = 12

Calculations:

$\%(C)=(11/12)\times100=91.67\%$	(1)

$\%(NC)=(1/12)\times100=8.33\%$	(2)

$\%(PC)=(0/12)\times100=0.00\%$	(3)

$\%(NV)=(0/12)\times100=0.00\%$	(4)

**Category: Management and Organizational Structure (MOS)**

**Subcategory: Recycling facility plan (RFP)**

Values: *C* = 12, *NC* = 0, *PC* = 0, *NV* = 0, *NI* = 12

Calculations:

$\%(C)=(12/12)\times100=100.00\%$	(1)

$\%(NC)=(0/12)\times100=0.00\%$	(2)

$\%(PC)=(0/12)\times100=0.00\%$	(3)

$\%(NV)=(0/12)\times100=0.00\%$	(4)

**Subcategory: Functional organizational chart and allocation of responsibilities (FOC)**

Values: *C* = 12, *NC* = 0, *PC* = 0, *NV* = 0, *NI* = 12

Calculations:

$\%(C)=(12/12)\times100=100.00\%$	(1)

$\%(NC)=(0/12)\times100=0.00\%$	(2)

$\%(PC)=(0/12)\times100=0.00\%$	(3)

$\%(NV)=(0/12)\times100=0.00\%$	(4)

**Subcategory: ISO certifications (IC)**

Values: *C* = 11, *NC* = 1, *PC* = 0, *NV* = 0, *NI* = 12

Calculations:

$\%(C)=(11/12)\times100=91.67\%$	(1)

$\%(NC)=(1/12)\times100=8.33\%$	(2)

$\%(PC)=(0/12)\times100=0.00\%$	(3)

$\%(NV)=(0/12)\times100=0.00\%$	(4)

**Category: Health, Safety and Environmental Performance (HSE)**

**Subcategory: Emergency response plan (ERP)**

Values: *C* = 12, *NC* = 0, *PC* = 0, *NV* = 0, *NI* = 12

Calculations:

$\%(C)=(12/12)\times100=100.00\%$	(1)

$\%(NC)=(0/12)\times100=0.00\%$	(2)

$\%(PC)=(0/12)\times100=0.00\%$	(3)

$\%(NV)=(0/12)\times100=0.00\%$	(4)

**Subcategory: Personal protective equipment (PPE)**

Values: *C* = 12, *NC* = 0, *PC* = 0, *NV* = 0, *NI* = 12

Calculations:

$\%(C)=(12/12)\times100=100.00\%$	(1)

$\%(NC)=(0/12)\times100=0.00\%$	(2)

$\%(PC)=(0/12)\times100=0.00\%$	(3)

$\%(NV)=(0/12)\times100=0.00\%$	(4)

**Subcategory: Soil environmental monitoring systems (SMS)**

Values: *C* = 7, *NC* = 0, *PC* = 5, *NV* = 0, *NI* = 12

Calculations:

$\%(C)=(7/12)\times100=58.33\%$	(1)

$\%(NC)=(0/12)\times100=0.00\%$	(2)

$\%(PC)=(5/12)\times100=41.67\%$	(3)

$\%(NV)=(0/12)\times100=0.00\%$	(4)

**Subcategory: Water environmental monitoring systems (WMS)**

Values: *C* = 10, *NC* = 0, *PC* = 2, *NV* = 0, *NI* = 12

Calculations:

$\%(C)=(10/12)\times100=83.33\%$	(1)

$\%(NC)=(0/12)\times100=0.00\%$	(2)

$\%(PC)=(2/12)\times100=16.67\%$	(3)

$\%(NV)=(0/12)\times100=0.00\%$	(4)

**Subcategory: Air environmental monitoring systems (AMS)**

Values: *C* = 11, *NC* = 0, *PC* = 1, *NV* = 0, *NI* = 12

Calculations:

$\%(C)=(11/12)\times100=91.67\%$	(1)

$\%(NC)=(0/12)\times100=0.00\%$	(2)

$\%(PC)=(1/12)\times100=8.33\%$	(3)

$\%(NV)=(0/12)\times100=0.00\%$	(4)

**Subcategory: Noise safety monitoring systems (NSM)**

Values: *C* = 11, *NC* = 0, *PC* = 1, *NV* = 0, *NI* = 12

Calculations:

$\%(C)=(11/12)\times100=91.67\%$	(1)

$\%(NC)=(0/12)\times100=0.00\%$	(2)

$\%(PC)=(1/12)\times100=8.33\%$	(3)

$\%(NV)=(0/12)\times100=0.00\%$	(4)

**Subcategory: Intertidal zone control (IZC)**

Values: *C* = 9, *NC* = 0, *PC* = 3, *NV* = 0, *NI* = 12

Calculations:

$\%(C)=(9/12)\times100=75.00\%$	(1)

$\%(NC)=(0/12)\times100=0.00\%$	(2)

$\%(PC)=(3/12)\times100=25.00\%$	(3)

$\%(NV)=(0/12)\times100=0.00\%$	(4)

**Subcategory: Regular medical surveillance (RMS)**

Values: *C* = 12, *NC* = 0, *PC* = 0, *NV* = 0, *NI* = 12

Calculations:

$\%(C)=(12/12)\times100=100.00\%$	(1)

$\%(NC)=(0/12)\times100=0.00\%$	(2)

$\%(PC)=(0/12)\times100=0.00\%$	(3)

$\%(NV)=(0/12)\times100=0.00\%$	(4)

**Subcategory: Training monitoring and documentation (TMD)**

Values: *C* = 12, *NC* = 0, *PC* = 0, *NV* = 0, *NI* = 12

Calculations:

$\%(C)=(12/12)\times100=100.00\%$	(1)

$\%(NC)=(0/12)\times100=0.00\%$	(2)

$\%(PC)=(0/12)\times100=0.00\%$	(3)

$\%(NV)=(0/12)\times100=0.00\%$	(4)

**Category: Recycling Operations (RO)**

**Subcategory: Ship reception process (SRS)**

Values: *C* = 12, *NC* = 0, *PC* = 0, *NV* = 0, *NI* = 12

Calculations:

$\%(C)=(12/12)\times100=100.00\%$	(1)

$\%(NC)=(0/12)\times100=0.00\%$	(2)

$\%(PC)=(0/12)\times100=0.00\%$	(3)

$\%(NV)=(0/12)\times100=0.00\%$	(4)

**Subcategory: Ship Recycling Plan (SRP)**

Values: *C* = 12, *NC* = 0, *PC* = 0, *NV* = 0, *NI* = 12

Calculations:

$\%(C)=(12/12)\times100=100.00\%$	(1)

$\%(NC)=(0/12)\times100=0.00\%$	(2)

$\%(PC)=(0/12)\times100=0.00\%$	(3)

$\%(NV)=(0/12)\times100=0.00\%$	(4)

**Subcategory: Cutting and safety procedures (CSP)**

Values: *C* = 12, *NC* = 0, *PC* = 0, *NV* = 0, *NI* = 12

Calculations:

$\%(C)=(12/12)\times100=100.00\%$	(1)

$\%(NC)=(0/12)\times100=0.00\%$	(2)

$\%(PC)=(0/12)\times100=0.00\%$	(3)

$\%(NV)=(0/12)\times100=0.00\%$	(4)

**Subcategory: Access and movement control (AMC)**

Values: *C* = 12, *NC* = 0, *PC* = 0, *NV* = 0, *NI* = 12

Calculations:

$\%(C)=(12/12)\times100=100.00\%$	(1)

$\%(NC)=(0/12)\times100=0.00\%$	(2)

$\%(PC)=(0/12)\times100=0.00\%$	(3)

$\%(NV)=(0/12)\times100=0.00\%$	(4)

**Category: Hazardous Waste and Materials Management (HWM)**

**Subcategory: Treatment of hazardous waste (THW)**

Values: *C* = 8, *NC* = 4, *PC* = 0, *NV* = 0, *NI* = 12

Calculations:

$\%(C)=(8/12)\times100=66.67\%$	(1)

$\%(NC)=(4/12)\times100=33.33\%$	(2)

$\%(PC)=(0/12)\times100=0.00\%$	(3)

$\%(NV)=(0/12)\times100=0.00\%$	(4)

**Subcategory: Treatment of non-hazardous waste (TNW)**

Values: *C* = 8, *NC* = 4, *PC* = 0, *NV* = 0, *NI* = 12

Calculations:

$\%(C)=(8/12)\times100=66.67\%$	(1)

$\%(NC)=(4/12)\times100=33.33\%$	(2)

$\%(PC)=(0/12)\times100=0.00\%$	(3)

$\%(NV)=(0/12)\times100=0.00\%$	(4)

**Subcategory: Documentation for waste traceability (DWT)**

Values: *C* = 12, *NC* = 0, *PC* = 0, *NV* = 0, *NI* = 12

Calculations:

$\%(C)=(12/12)\times100=100.00\%$	(1)

$\%(NC)=(0/12)\times100=0.00\%$	(2)

$\%(PC)=(0/12)\times100=0.00\%$	(3)

$\%(NV)=(0/12)\times100=0.00\%$	(4)

**Subcategory: Temporary storage (TS)**

Values: *C* = 12, *NC* = 0, *PC* = 0, *NV* = 0, *NI* = 12

Calculations:

$\%(C)=(12/12)\times100=100.00\%$	(1)

$\%(NC)=(0/12)\times100=0.00\%$	(2)

$\%(PC)=(0/12)\times100=0.00\%$	(3)

$\%(NV)=(0/12)\times100=0.00\%$	(4)

**Subcategory: Transport oversight and tracking (TOT)**

Values: *C* = 12, *NC* = 0, *PC* = 0, *NV* = 0, *NI* = 12

Calculations:

$\%(C)=(12/12)\times100=100.00\%$	(1)

$\%(NC)=(0/12)\times100=0.00\%$	(2)

$\%(PC)=(0/12)\times100=0.00\%$	(3)

$\%(NV)=(0/12)\times100=0.00\%$	(4)

**Category: Indicators and Monitoring (IM)**

**Subcategory: Record of accidents, incidents, and occupational diseases (AID)**

Values: *C* = 10, *NC* = 1, *PC* = 1, *NV* = 0, *NI* = 12

Calculations:

$\%(C)=(10/12)\times100=83.33\%$	(1)

$\%(NC)=(1/12)\times100=8.33\%$	(2)

$\%(PC)=(1/12)\times100=8.33\%$	(3)

$\%(NV)=(0/12)\times100=0.00\%$	(4)

**Subcategory: Progress reports (internal or client-mandated) (PR)**

Values: *C* = 6, *NC* = 5, *PC* = 0, *NV* = 1, *NI* = 12

Calculations:

$\%(C)=(6/12)\times100=50.00\%$	(1)

$\%(NC)=(5/12)\times100=41.67\%$	(2)

$\%(PC)=(0/12)\times100=0.00\%$	(3)

$\%(NV)=(1/12)\times100=8.33\%$	(4)

**Subcategory: Performance indicators (KPIs) (PI)**

Values: *C* = 6, *NC* = 5, *PC* = 1, *NV* = 0, *NI* = 12

Calculations:

$\%(C)=(6/12)\times100=50.00\%$	(1)

$\%(NC)=(5/12)\times100=41.67\%$	(2)

$\%(PC)=(1/12)\times100=8.33\%$	(3)

$\%(NV)=(0/12)\times100=0.00\%$	(4)

**Calculation of percentages by parameter for subcategories - Facilities not included in the EU List**

Definitions:

*C* = Compliant

*NC* = Non-compliant

*PC* = Partially compliant

*NV* = Not validated

*NI* = Number of installations analysed

$\%(C)=(C/NI)\times100$	(1)

$\%(NC)=(NC/NI)\times100$	(2)

$\%(PC)=(PC/NI)\times100$	(3)

$\%(NV)=(NV/NI)\times100$	(4)

**Category: Legal Authorization and Regulatory Compliance (LAC)**

**Subcategory: Approval by competent authorities (ACA)**

Values: *C* = 9, *NC* = 1, *PC* = 0, *NV* = 0, *NI* = 10

Calculations:

$\%(C)=(9/10)\times100=90.00\%$	(1)

$\%(NC)=(1/10)\times100=10.00\%$	(2)

$\%(PC)=(0/10)\times100=0.00\%$	(3)

$\%(NV)=(0/10)\times100=0.00\%$	(4)

**Subcategory: Environmental license (EL)**

Values: *C* = 9, *NC* = 1, *PC* = 0, *NV* = 0, *NI* = 10

Calculations:

$\%(C)=(9/10)\times100=90.00\%$	(1)

$\%(NC)=(1/10)\times100=10.00\%$	(2)

$\%(PC)=(0/10)\times100=0.00\%$	(3)

$\%(NV)=(0/10)\times100=0.00\%$	(4)

**Subcategory: Operational license (OL)**

Values: *C* = 10, *NC* = 0, *PC* = 0, *NV* = 0, *NI* = 10

Calculations:

$\%(C)=(10/10)\times100=100.00\%$	(1)

$\%(NC)=(0/10)\times100=0.00\%$	(2)

$\%(PC)=(0/10)\times100=0.00\%$	(3)

$\%(NV)=(0/10)\times100=0.00\%$	(4)

**Category: Project Design and Infrastructure (PI)**

**Subcategory: Dismantling method (DM)**

Values: *C* = 10, *NC* = 0, *PC* = 0, *NV* = 0, *NI* = 10

Calculations:

$\%(C)=(10/10)\times100=100.00\%$	(1)

$\%(NC)=(0/10)\times100=0.00\%$	(2)

$\%(PC)=(0/10)\times100=0.00\%$	(3)

$\%(NV)=(0/10)\times100=0.00\%$	(4)

**Subcategory: Presence of impermeable flooring with drainage systems (PIF)**

Values: *C* = 9, *NC* = 1, *PC* = 0, *NV* = 0, *NI* = 10

Calculations:

$\%(C)=(9/10)\times100=90.00\%$	(1)

$\%(NC)=(1/10)\times100=10.00\%$	(2)

$\%(PC)=(0/10)\times100=0.00\%$	(3)

$\%(NV)=(0/10)\times100=0.00\%$	(4)

**Subcategory: Primary and secondary cutting areas (PSC)**

Values: *C* = 9, *NC* = 0, *PC* = 0, *NV* = 1, *NI* = 10

Calculations:

$\%(C)=(9/10)\times100=90.00\%$	(1)

$\%(NC)=(0/10)\times100=0.00\%$	(2)

$\%(PC)=(0/10)\times100=0.00\%$	(3)

$\%(NV)=(1/10)\times100=10.00\%$	(4)

**Subcategory: Waste storage facilities (WSF)**

Values: *C* = 10, *NC* = 0, *PC* = 0, *NV* = 0, *NI* = 10

Calculations:

$\%(C)=(10/10)\times100=100.00\%$	(1)

$\%(NC)=(0/10)\times100=0.00\%$	(2)

$\%(PC)=(0/10)\times100=0.00\%$	(3)

$\%(NV)=(0/10)\times100=0.00\%$	(4)

**Category: Management and Organizational Structure (MOS)**

**Subcategory: Recycling facility plan (RFP)**

Values: *C* = 9, *NC* = 0, *PC* = 0, *NV* = 1, *NI* = 10

Calculations:

$\%(C)=(9/10)\times100=90.00\%$	(1)

$\%(NC)=(0/10)\times100=0.00\%$	(2)

$\%(PC)=(0/10)\times100=0.00\%$	(3)

$\%(NV)=(1/10)\times100=10.00\%$	(4)

**Subcategory: Functional organizational chart and allocation of responsibilities (FOC)**

Values: *C* = 9, *NC* = 0, *PC* = 0, *NV* = 1, *NI* = 10

Calculations:

$\%(C)=(9/10)\times100=90.00\%$	(1)

$\%(NC)=(0/10)\times100=0.00\%$	(2)

$\%(PC)=(0/10)\times100=0.00\%$	(3)

$\%(NV)=(1/10)\times100=10.00\%$	(4)

**Subcategory: ISO certifications (IC)**

Values: *C* = 7, *NC* = 1, *PC* = 0, *NV* = 2, *NI* = 10

Calculations:

$\%(C)=(7/10)\times100=70.00\%$	(1)

$\%(NC)=(1/10)\times100=10.00\%$	(2)

$\%(PC)=(0/10)\times100=0.00\%$	(3)

$\%(NV)=(2/10)\times100=20.00\%$	(4)

**Category: Health, Safety and Environmental Performance (HSE)**

**Subcategory: Emergency response plan (ERP)**

Values: *C* = 10, *NC* = 0, *PC* = 0, *NV* = 0, *NI* = 10

Calculations:

$\%(C)=(10/10)\times100=100.00\%$	(1)

$\%(NC)=(0/10)\times100=0.00\%$	(2)

$\%(PC)=(0/10)\times100=0.00\%$	(3)

$\%(NV)=(0/10)\times100=0.00\%$	(4)

**Subcategory: Personal protective equipment (PPE)**

Values: *C* = 10, *NC* = 0, *PC* = 0, *NV* = 0, *NI* = 10

Calculations:

$\%(C)=(10/10)\times100=100.00\%$	(1)

$\%(NC)=(0/10)\times100=0.00\%$	(2)

$\%(PC)=(0/10)\times100=0.00\%$	(3)

$\%(NV)=(0/10)\times100=0.00\%$	(4)

**Subcategory: Soil environmental monitoring systems (SMS)**

Values: *C* = 4, *NC* = 2, *PC* = 4, *NV* = 0, *NI* = 10

Calculations:

$\%(C)=(4/10)\times100=40.00\%$	(1)

$\%(NC)=(2/10)\times100=20.00\%$	(2)

$\%(PC)=(4/10)\times100=40.00\%$	(3)

$\%(NV)=(0/10)\times100=0.00\%$	(4)

**Subcategory: Water environmental monitoring systems (WMS)**

Values: *C* = 4, *NC* = 1, *PC* = 5, *NV* = 0, *NI* = 10

Calculations:

$\%(C)=(4/10)\times100=40.00\%$	(1)

$\%(NC)=(1/10)\times100=10.00\%$	(2)

$\%(PC)=(5/10)\times100=50.00\%$	(3)

$\%(NV)=(0/10)\times100=0.00\%$	(4)

**Subcategory: Air environmental monitoring systems (AMS)**

Values: *C* = 5, *NC* = 2, *PC* = 3, *NV* = 0, *NI* = 10

Calculations:

$\%(C)=(5/10)\times100=50.00\%$	(1)

$\%(NC)=(2/10)\times100=20.00\%$	(2)

$\%(PC)=(3/10)\times100=30.00\%$	(3)

$\%(NV)=(0/10)\times100=0.00\%$	(4)

**Subcategory: Noise safety monitoring systems (NSM)**

Values: *C* = 6, *NC* = 4, *PC* = 0, *NV* = 0, *NI* = 10

Calculations:

$\%(C)=(6/10)\times100=60.00\%$	(1)

$\%(NC)=(4/10)\times100=40.00\%$	(2)

$\%(PC)=(0/10)\times100=0.00\%$	(3)

$\%(NV)=(0/10)\times100=0.00\%$	(4)

**Subcategory: Intertidal zone control (IZC)**

Values: *C* = 5, *NC* = 2, *PC* = 3, *NV* = 0, *NI* = 10

Calculations:

$\%(C)=(5/10)\times100=50.00\%$	(1)

$\%(NC)=(2/10)\times100=20.00\%$	(2)

$\%(PC)=(3/10)\times100=30.00\%$	(3)

$\%(NV)=(0/10)\times100=0.00\%$	(4)

**Subcategory: Regular medical surveillance (RMS)**

Values: *C* = 7, *NC* = 3, *PC* = 0, *NV* = 0, *NI* = 10

Calculations:

$\%(C)=(7/10)\times100=70.00\%$	(1)

$\%(NC)=(3/10)\times100=30.00\%$	(2)

$\%(PC)=(0/10)\times100=0.00\%$	(3)

$\%(NV)=(0/10)\times100=0.00\%$	(4)

**Subcategory: Training monitoring and documentation (TMD)**

Values: *C* = 6, *NC* = 0, *PC* = 4, *NV* = 0, *NI* = 10

Calculations:

$\%(C)=(6/10)\times100=60.00\%$	(1)

$\%(NC)=(0/10)\times100=0.00\%$	(2)

$\%(PC)=(4/10)\times100=40.00\%$	(3)

$\%(NV)=(0/10)\times100=0.00\%$	(4)

**Category: Recycling Operations**

**Subcategory: Ship reception process (SRS)**

Values: *C* = 10, *NC* = 0, *PC* = 0, *NV* = 0, *NI* = 10

Calculations:

$\%(C)=(10/10)\times100=100.00\%$	(1)

$\%(NC)=(0/10)\times100=0.00\%$	(2)

$\%(PC)=(0/10)\times100=0.00\%$	(3)

$\%(NV)=(0/10)\times100=0.00\%$	(4)

**Subcategory: Ship Recycling Plan (SRP)**

Values: *C* = 9, *NC* = 0, *PC* = 0, *NV* = 1, *NI* = 10

Calculations:

$\%(C)=(9/10)\times100=90.00\%$	(1)

$\%(NC)=(0/10)\times100=0.00\%$	(2)

$\%(PC)=(0/10)\times100=0.00\%$	(3)

$\%(NV)=(1/10)\times100=10.00\%$	(4)

**Subcategory: Cutting and safety procedures (CSP)**

Values: *C* = 9, *NC* = 0, *PC* = 0, *NV* = 1, *NI* = 10

Calculations:

$\%(C)=(9/10)\times100=90.00\%$	(1)

$\%(NC)=(0/10)\times100=0.00\%$	(2)

$\%(PC)=(0/10)\times100=0.00\%$	(3)

$\%(NV)=(1/10)\times100=10.00\%$	(4)

**Subcategory: Access and movement control (AMC)**

Values: *C* = 9, *NC* = 1, *PC* = 0, *NV* = 0, *NI* = 10

Calculations:

$\%(C)=(9/10)\times100=90.00\%$	(1)

$\%(NC)=(1/10)\times100=10.00\%$	(2)

$\%(PC)=(0/10)\times100=0.00\%$	(3)

$\%(NV)=(0/10)\times100=0.00\%$	(4)

**Category: Hazardous Waste and Materials Management (HWM)**

**Subcategory: Treatment of hazardous waste (THW)**

Values: *C* = 6, *NC* = 4, *PC* = 0, *NV* = 0, *NI* = 10

Calculations:

$\%(C)=(6/10)\times100=60.00\%$	(1)

$\%(NC)=(4/10)\times100=40.00\%$	(2)

$\%(PC)=(0/10)\times100=0.00\%$	(3)

$\%(NV)=(0/10)\times100=0.00\%$	(4)

**Subcategory: Treatment of non-hazardous waste (TNW)**

Values: *C* = 7, *NC* = 3, *PC* = 0, *NV* = 0, *NI* = 10

Calculations:

$\%(C)=(7/10)\times100=70.00\%$	(1)

$\%(NC)=(3/10)\times100=30.00\%$	(2)

$\%(PC)=(0/10)\times100=0.00\%$	(3)

$\%(NV)=(0/10)\times100=0.00\%$	(4)

**Subcategory: Documentation for waste traceability (DWT)**

Values: *C* = 9, *NC* = 1, *PC* = 0, *NV* = 0, *NI* = 10

Calculations:

$\%(C)=(9/10)\times100=90.00\%$	(1)

$\%(NC)=(1/10)\times100=10.00\%$	(2)

$\%(PC)=(0/10)\times100=0.00\%$	(3)

$\%(NV)=(0/10)\times100=0.00\%$	(4)

**Subcategory: Temporary storage (TS)**

Values: *C* = 10, *NC* = 0, *PC* = 0, *NV* = 0, *NI* = 10

Calculations:

$\%(C)=(10/10)\times100=100.00\%$	(1)

$\%(NC)=(0/10)\times100=0.00\%$	(2)

$\%(PC)=(0/10)\times100=0.00\%$	(3)

$\%(NV)=(0/10)\times100=0.00\%$	(4)

**Subcategory: Transport oversight and tracking (TOT)**

Values: *C* = 10, *NC* = 0, *PC* = 0, *NV* = 0, *NI* = 10

Calculations:

$\%(C)=(10/10)\times100=100.00\%$	(1)

$\%(NC)=(0/10)\times100=0.00\%$	(2)

$\%(PC)=(0/10)\times100=0.00\%$	(3)

$\%(NV)=(0/10)\times100=0.00\%$	(4)

**Category: Indicators and Monitoring (IM)**

**Subcategory: Record of accidents, incidents, and occupational diseases (AID)**

Values: *C* = 7, *NC* = 3, *PC* = 0, *NV* = 0, *NI* = 10

Calculations:

$\%(C)=(7/10)\times100=70.00\%$	(1)

$\%(NC)=(3/10)\times100=30.00\%$	(2)

$\%(PC)=(0/10)\times100=0.00\%$	(3)

$\%(NV)=(0/10)\times100=0.00\%$	(4)

**Subcategory: Progress reports (internal or client-mandated) (PR)**

Values: *C* = 8, *NC* = 2, *PC* = 0, *NV* = 0, *NI* = 10

Calculations:

$\%(C)=(8/10)\times100=80.00\%$	(1)

$\%(NC)=(2/10)\times100=20.00\%$	(2)

$\%(PC)=(0/10)\times100=0.00\%$	(3)

$\%(NV)=(0/10)\times100=0.00\%$	(4)

**Subcategory: Performance indicators (KPIs) (PI)**

Values: *C* = 5, *NC* = 1, *PC* = 4, *NV* = 0, *NI* = 10

Calculations:

$\%(C)=(5/10)\times100=50.00\%$	(1)

$\%(NC)=(1/10)\times100=10.00\%$	(2)

$\%(PC)=(4/10)\times100=40.00\%$	(3)

$\%(NV)=(0/10)\times100=0.00\%$	(4)

**Calculation of percentages by category for facilities included and not included in the EU list**

The averages of the parameters for each group of subcategories listed above in this document were calculated. For each category, the absolute values of C, NC, PC, and NV from all subcategories were summed, and the percentages were then calculated relative to the total number of facilities in each subcategory group.

General formulas:

$\%(C)=(\Sigma C/\Sigma NI)\times100$	(1)

$\%(NC)=(\Sigma NC/\Sigma NI)\times100$	(2)

$\%(PC)=(\Sigma PC/\Sigma NI)\times100$	(3)

$\%(NV)=(\Sigma NV/\Sigma NI)\times100$	(4)

**Category: Legal Authorization and Regulatory Compliance (LAC)**

For facilities included in the EU List:
%(*C*) = 100.00%	(1)
%(*NC*) = 0.00%	(2)
%(*PC*) = 0.00%	(3)
%(*NV*) = 0.00%	(4)

For facilities not included in the EU List:
%(*C*) = 93.33%	(1)
%(*NC*) = 6.67%	(2)
%(*PC*) = 0.00%	(3)
%(*NV*) = 0.00%	(4)

**Category: Project Design and Infrastructure (PI)**

For facilities included in the EU List:
%(*C*) = 97.92%	(1)
%(*NC*) = 2.08%	(2)
%(*PC*) = 0.00%	(3)
%(*NV*) = 0.00%	(4)

For facilities not included in the EU List:
%(*C*) = 95.00%	(1)
%(*NC*) = 2.50%	(2)
%(*PC*) = 0.00%	(3)
%(*NV*) = 2.50%	(4)

**Category: Management and Organizational Structure (MOS)**

For facilities included in the EU List:
%(*C*) = 97.22%	(1)
%(*NC*) = 2.78%	(2)
%(*PC*) = 0.00%	(3)
%(*NV*) = 0.00%	(4)

For facilities not included in the EU List:
%(*C*) = 83.33%	(1)
%(*NC*) = 3.33%	(2)
%(*PC*) = 0.00%	(3)
%(*NV*) = 13.33%	(4)

**Category: Health, Safety and Environmental Performance (HSE)**

For facilities included in the EU List:
%(*C*) = 88.89%	(1)
%(*NC*) = 0.00%	(2)
%(*PC*) = 11.11%	(3)
%(*NV*) = 0.00%	(4)

For facilities not included in the EU List:
%(*C*) = 63.33%	(1)
%(*NC*) = 15.56%	(2)
%(*PC*) = 21.11%	(3)
%(*NV*) = 0.00%	(4)

**Category: Recycling Operations (RO)**

For facilities included in the EU List:
%(*C*) = 100.00%	(1)
%(*NC*) = 0.00%	(2)
%(*PC*) = 0.00%	(3)
%(*NV*) = 0.00%	(4)

For facilities not included in the EU List:
%(*C*) = 92.50%	(1)
%(*NC*) = 2.50%	(2)
%(*PC*) = 0.00%	(3)
%(*NV*) = 5.00%	(4)

**Category: Hazardous Waste and Materials Management (HWM)**

For facilities included in the EU List:
%(*C*) = 86.67%	(1)
%(*NC*) = 13.33%	(2)
%(*PC*) = 0.00%	(3)
%(*NV*) = 0.00%	(4)

For facilities not included in the EU List:
%(*C*) = 84.00%	(1)
%(*NC*) = 16.00%	(2)
%(*PC*) = 0.00%	(3)
%(*NV*) = 0.00%	(4)

**Category: Indicators and Monitoring (IM)**

For facilities included in the EU List:
%(*C*) = 61.11%	(1)
%(*NC*) = 30.55%	(2)
%(*PC*) = 5.56%	(3)
%(*NV*) = 2.78%	(4)

For facilities not included in the EU List:
%(*C*) = 66.67%	(1)
%(*NC*) = 20.00%	(2)
%(*PC*) = 13.33%	(3)
%(*NV*) = 0.00%	(4)

**Category: Overall Average**

The overall average was calculated using the arithmetic mean of the percentages for each parameter in each category.

For facilities included in the EU List:

$\%(C)=(100+97.92+97.22+88.89+100+86.67+61.11)/7=90.26$%	(1)

$\%(NC)=(0 +2.08+2.78+0+0+13.33+30.55)/7=6.96\%$	(2)

$\%(PC)=(0+0+0+11.11+0+0+5.56)/7=2.38\%$	(3)

$\%(NV)=(0+0+0+0+0+0+2.78)/7=0.40\%$	(4)

For facilities not included in the EU List:

$\%(C)=(93.33+95+83.33+63.33+92.5+84+66.67)/7=82.59\%$	(1)

$\%(NC)=(6.67+2.5+3.33+15.56+2.5+16+20)/7=9.51\%$	(2)

$\%(PC)=(0+0+0+21.11+0+0+13.33)/7=4.92\%$	(3)

$\%(NV)=(0+2.5+13.33+0+5+0+0)/7=2.98\%$	(4)
